# Camptothecin bioprocessing from *Aspergillus terreus*, an endophyte of *Catharanthus roseus*: antiproliferative activity, topoisomerase inhibition and cell cycle analysis

**DOI:** 10.1186/s12934-023-02270-4

**Published:** 2024-01-05

**Authors:** Ashraf S. A. El-Sayed, Abdelaleim I. ElSayed, Khalid M. Wadan, Sayed S. El-Saadany, Nouran A. A. Abd El-Hady

**Affiliations:** 1 Enzymology and Fungal Biotechnology Lab, Botany and Microbiology Department, Faculty of Science, Zagazig University, Zagazig, 44519 Egypt; 2 Biochemistry Department, Faculty of Agriculture, Zagazig University, Zagazig, 44519 Egypt

**Keywords:** Camptothecin, *Aspergillus terreus*, *Catharanthus roseus*, Anticancer activity, Topoisomerase inhibitors, Apoptosis, Cell cycle, Wound healing

## Abstract

Attenuation of camptothecin (CPT) productivity by fungi with preservation and subculturing is the challenge that halts fungi to be an industrial platform of CPT production. Thus, screening for novel endophytic fungal isolates with metabolic stability for CPT production was the objective. *Catharanthus roseus* is one of the medicinal plants with diverse bioactive metabolites that could have a plethora of novel endophytes with unique metabolites. Among the endophytes of *C. roseus*, *Aspergillus terreus* EFBL-NV OR131583.1 had the most CPT producing potency (90.2 μg/l), the chemical identity of the putative CPT was verified by HPLC, FT-IR, NMR and LC–MS/MS. The putative *A. terreus* CPT had the same molecular mass (349 m*/z*), and molecular fragmentation patterns of the authentic one, as revealed from the MS/MS analyses. The purified CPT had a strong activity against MCF7 (5.27 μM) and UO-31 (2.2 μM), with a potential inhibition to Topo II (IC_50_ value 0.52 nM) than Topo 1 (IC_50_ value 6.9 nM). The CPT displayed a high wound healing activity to UO-31 cells, stopping their metastasis, matrix formation and cell immigration. The purified CPT had a potential inducing activity to the cellular apoptosis of UO-31 by ~ 17 folds, as well as, arresting their cellular division at the S-phase, compared to the control cells. Upon Plackett–Burman design, the yield of CPT by *A. terreus* was increased by ~ 2.6 folds, compared to control. The yield of CPT by *A. terreus* was sequentially suppressed with the fungal storage and subculturing, losing ~ 50% of their CPT productivity by 3rd month and 5th generation. However, the productivity of the attenuated *A. terreus* culture was completely restored by adding 1% surface sterilized leaves of *C. roseus,* and the CPT yield was increased over-the-first culture by ~ 3.2 folds (315.2 μg/l)*.* The restoring of CPT productivity of *A. terreus* in response to indigenous microbiome of *C. roseus*, ensures the *A. terreus*-microbiome interactions, releasing a chemical signal that triggers the CPT productivity of *A. terreus*. This is the first reports exploring the potency of *A. terreus,* endophyte of *C. roseus*” to be a platform for industrial production of CPT, with an affordable sustainability with addition of *C. roseus* microbiome.

**Supplementary Information:**

The online version contains supplementary material available at 10.1186/s12934-023-02270-4.

## Introduction

Camptothecin (CPT) is a quinoline pentacyclic alkaloid that was firstly isolated from *Camptotheca acuminata* (Nyssaceae, happy tree), in the southwest provinces of China [1]. CPT displayed a powerful broad-range antiproliferative activity towards various types of solid tumors [2]. CPT derivatives are one of the most prescribed anticancer drugs, after Taxol and vincristine [3]. The powerful antiproliferative activity of CPT derivatives elaborates from their higher affinity to bind with the topoisomerase I and II [4], causing a subsequent inhibition to the enzyme activity. DNA topoisomerases exists in all nucleated cells maintaining the topology of DNA strands during DNA replication, RNA transcription, recombination, chromatin association and remodeling [4–6]. The topoisomerase I breaks only one strand of duplex DNA giving 3′-phospho-tyrosine intermediate, type II breaks both strands of DNA duplex with the formation of a pair of 5′-phosphotyrosine covalent intermediates [4]. The topoisomerase causes single/double strand breakages, relaxing the DNA strands, and catalyzes the religation of the cleaved DNA [4]. In presence of CPT, the DNA-topoisomerase I, II complex was stabilized, preventing the subsequent step of religation and cleavage [7–10]. CPT is one of the monoterpenoid indole alkaloids (TIA) that derived from the strictosidine precursor, which is produced by the combination of monoterpenoid secologanin and tryptamine indole amino acid [11, 12].

*Camptotheca acuminata* is the major source of CPT, the seeds and bark of this plant contains ~ 0.2–0.3%, whereas the leaves contains up to 0.4% of CPT [13–15]. The bark of *C. acuminata* contains about 0.5% of 10-hydroxycamptothecin [13]. However, the tiny yield, with the heavy demand of this compound, causing a drastic harvesting of this plant, with subsequent negative effect on their natural ecosystem, in addition to the limitation of this plant to certain geographical niches [16–18]. Additionally, the natural low abundance, diverse aromaticity and complexity of extraction of CPT from plants are the major challenges [19, 20]. Endophytic fungi from the medicinal plants were considered as an unexploited reservoir of numerous secondary metabolites with diverse activity that could be due to the horizontal gene transfer, sharing the diverse molecular biosynthetic machineries of the plant host and their endogenous microbiome [21–25]. The metabolic potency of fungi for CPT biosynthesis has been firstly emphasized with the ability of *Entrophospora infrequens* an endophyte of *Nothapodytes foetida* [26–28] for CPT production followed by a plethora of endophytic fungi from various plants with the ability to produce CPT were reported [21–25]. The metabolic biosynthetic potency of CPT by the endophytic fungi elevates the prospective industrial applications of fungi, for their fast growth, feasibility of bulk biomass production and independence on the environmental conditions [26, 27, 29]. However, the further endeavor for employment of fungi for the commercial application of CPT is the attenuation of the biosynthetic machinery of CPT, with an obvious subsequent reduction to the CPT yield with the fungal storage and subculturing [14, 26–30]. Several trials have been implemented to restore the biosynthetic potency of fungi via co-cultivation with the microbiome of the host plant, addition of different plant extracts. The yield of CPT by *Aspergillus terreus*, an endophyte of *Ficus elastica* [23], *Cestrum parqui* [21], *Cinnamomum camphora* [22], *A. flavus,* an endophyte of *Astragalus fruticosus* [25], *Penicillium chrysogenum* an endozoic of *Cliona* sp [24] was strongly reduced with the fungal storage. Thus, the main objective of this study was to isolate a novel endophytic fungal isolate with a plausible CPT biosynthetic stability and to assess their antiproliferative, biological activity, and topoisomerases inhibition activity.

## Materials and methods

### Collection of the plant samples and isolation of endophytic fungi

*Catharanthus roseus* were collected from the Botanical Garden of Zagazig University, Zagazig, Alsharqia province, Egypt, in September/2021. Fresh parts including leaves, stems and flowers were brought to the lab in sterile plastic bags, washed thoroughly with sterile distilled, sectioned into small segments (1 × 1 cm). The plant parts were surface sterilized with 70% ethyl alcohol for 1 min, 2.5% sodium hypochlorite for 2 min, then washed with sterile distilled water to exclude any epiphytic microbial flora [31, 32]. The sterilized plant segments were placed on the surface of potato dextrose agar (PDA), and Czapek’s-Dox media with antibacterial agent ampicillin (1 μg/ml) and incubated for 8 days at 30 °C [33, 34]. The recovered fungal isolates were morphologically identified according to macroscopical and microscopical features according to the universal keys of fungal identification [35–40].

### Molecular identification of the isolated endophytic fungi

The potent CPT producing fungal isolate were molecularly confirmed based on their ITS1-ITS2 sequences [31, 41, 42]. The genomic DNA (gDNA) was extracted by cetyltrimethylammonium bromide (CTAP) reagent, used as a PCR template with the primers ITS4 5′-GGAAGTAAAAGTC-GTAACAAGG-3′ and ITS5 5′-TCCTCCGCTTATTGATATGC-3′. The PCR reaction contains 10 μl of 2 × PCR master mixture (i-Taq™, Cat. No. 25027, INTRON Biotech), 2 μl of the gDNA, 1 μl of each primer (10 pmol), and completed to 20 μl with sterile distilled water. The PCR was programmed to initial denaturation at 94 °C for 2 min, then denaturation at 94 °C for 30 s, annealing at 55 °C for 10 s, extension at 72 °C for 30 s for 35 cycles, and final extension at 72 °C for 2 min. The PCR products were analyzed by 1.5% agarose gel in TBE buffer, sequenced by Applied Biosystems Sequencer, HiSQV Bases, Version 6.0 with the same primers. The sequences were non-redundantly searched on BLAST tool, aligned by ClustalW muscle algorithm [43], and the phylogenetic relationship was constructed with neighbor-joining method with 100 bootstrap replication [44].

### Screening, chromatographic analyses of CPT production by the recovered fungi

The CPT productivity by the recovered endophytic fungal isolates was screened by growing on potato dextrose broth (PDB) (BD, Difco, Cat# DF0549-17-9) [22, 23, 45, 46]. A agar plug from 6 days old PDA fungal culture was taken from each fungus, inoculated into 50 ml PDB/250 ml Erlenmeyer flask, incubated at 30 °C for 15 days, the cultures were filtered and the CPT was extracted from the fungal filtrates by methylene chloride, and concentrated by rotary evaporator till oily residues. The extract was fractionated by TLC (Merck 1 mm (20 × 20 cm), pre-coated silica gel plates, Silica gel 60 F254, KGaA, Darm. Germany) with dichloromethane and methanol (9:1 v/v), as solvent system. After running, the CPT spot was detected by illumination at λ_254_ nm, normalized to standard one (Cat. 7689-03-4). The putative CPT spots gave the same blue color, and relative mobility as standard was considered. The intensity of putative spots was determined by the Image J package, regarding to the known concentrations of authentic CPT. CPT was extracted from the spots of CPT containing silica [21–23] and analyzed by HPLC (YOUNG In, Chromass) with RP-C18 column (Cat. #959963-902) with methanol/ water (60:40 v/v) at a flow rate 1.0 ml/min, for 20 min. The CPT concentrations were assessed from the retention time and area of the peak at λ_360_ nm, compared to the authentic CPT [21–25]. The chemical identity were confirmed from the standard CPT retention time and peak area compared to the authentic one.

### UV–Vis, FT-IR, NMR, LC–MS/MS analyses

The purified putative CPT samples were dissolved in methanol and scanned by UV–Vis at wavelength range λ_200_-λ_500_ nm (RIGOL, Ultra-3000 Spectrophotometer). Methanol was used as blank baseline. Authentic CPT was scanned at the same conditions, and the spectroscopic identity of the sample was assigned comparing to the authentic one.

The FT-IR spectra of the sample of CPT were assessed from 400 to 4000 cm^−1^ with KBr discs, compared to authentic CPT. The chemical identity of the extracted CPT was resolved from the ^1^HNMR (JEOL, ECA-500II) [21–23]. The chemical shifts (δ-scale) and coupling constants (Hz) were expressed by ppm.

The chemical identity of the CPT samples was analyzed by the liquid chromatography-tandem mass spectrometry (LC–MS/MS) (Thermo Scientific LCQ Deca mass spectrometer, equipped with an electrospray source operated in positive ion mode) [23]. The mobile phases consisted of water with 0.1% formic acid (A), and acetonitrile with 0.1% formic acid (B). The sample were injected into a Thermo Scientific Hypersil Gold aQ (C18 column), and the elution system was a gradient of 2–98% mobile phase B over 30 min, with a flow rate of 0.2 ml/min, for total run time was 40 min. The electrospray ionization (ESI) source operated with a spray voltage of 4 kV and a capillary temperature of 250 °C. The ion trap was scanned in a positive-ion mode from m/z 300–2000, with recorded mass scan between 300 and 2000 Da. The chemical identity of the components was identified based on their mass spectra fragmentation pattern and retention times. Further fragmentation analyses to the selected peaks of the putative molecular mass corresponding to authentic CPT at 349.1 m/z. The identity of the extracted CPT was confirmed from the molecular fragmentation pattern corresponding to the authentic one.

### Antifungal activity guided-assay of the putative CPT samples

The activity of the extracted CPT from the selected fungal isolates was assessed towards various CPT and non-CPT producing fungal isolates recovered from the flowers of *Catharanthus roseus.* Different concentrations of the putative CPT extracts of the selected fungal isolates were injected into 9 mm wells on PDA culture plates of the recovered endophytic fungal isolates from *C. roseus*. The plates were incubated at 30 °C for 5 days, and the diameters of the inhibition zones were measured compared to 1% DMSO as negative control.

### Antiproliferative activity of the extracted CPT

The activity of the extracted CPT was assessed against breast carcinoma (MCF7) and Renal cancer cell lines (UO-31), compared to the normal oral epithelial cells (OEC), with MTT assay [47]. The breast carcinoma (MCF7) (ATCC HTB-22) and the Renal cancer (UO-31) (EZT-UO31-1) cell lines were obtained from the American Type Culture Collection and EZ-Biosystems. The cells were cultured on DMEM (Invitrogen/Life Technol.) supplemented with 10% FBS (Hyclone), 10 μg/ml of insulin (Sigma), and 50 U/ml penicillin and 50 μg/ml streptomycin. All of the other chemicals and reagents were from Sigma, or Invitrogen. The 96-well microtiter plate was seeded with 10^3^/well, incubated overnight at 37 °C, amended with various concentrations (1.0, 2.0, 4.0, 8.0 and 10 μM) of the purified CPT dissolved in 2% DMSO as vehicle, then further incubated for 48 h at the same conditions. DMSO at 2% was used as negative control The MTT reagent was added, the developed formazan complex with purple color was measured at λ_570_ nm. The IC_50_ value was expressed by the amount of CPT reducing the growth of tumor cells by about 50%, compared to the controls (without drug).

### Kinetics of DNA topoisomerase I inhibition in response to the extracted CPT

The human topoisomerase I activity was assessed based on converting of the supercoiled circular DNA into relaxed DNA [10], the relaxed DNA suppresses the fluorescent intensity than the supercoiled one of the fluorescence dye H19 (Cat.#. HRA020K, ProFoldin, Hu, USA). The reaction mixture of Topo I assay contains HT buffer, 10 × supercoiled plasmid DNA, 1500 × Dye H19 and 550 μl of 10 × H19 dilution buffer, incubated for 60 min at room temperature, in presence of different concentrations of the CPT. One unit is the enzyme activity was represented by the amount of enzyme required for relaxing of supercoiled DNA in 30 min at 37 ºC, the florescence emission intensity was measured at λ_535_ nm at excitation λ_485_ nm [8].

### Wound healing of tumor cells in response to the extracted CPT

The wound healing and cell migration potency of the tested tumor cells in response to the extracted CPT was assessed [48, 49]. Breifly, the UO-31 cells were seeded at 5 × 10^4^ cells per 40 mm^2^ plate, incubated for 24 h to form a confluent monolayer (about 60 k/cm^2^), then a wound/ scratch was made. The plate were rinsed with PBS and treated with the extract of CPT. DMSO was used as control. The wound closure due to the cell migration was monitored, imaged by phase-contrast microscope. The wound healing percentage was determined based on the gap area of the treated cells, compared to the control cells.

### Apoptosis and cell cycle analyses of UO-31 cells in response the extracted CPT

The apoptosis of the UO-31 cells was detected using Annexin V-FITC Apoptosis Kit (Cat #: K101-25) according to the manufacturer’s instructions. The concept of this assay is relied on, with the initiation of apoptosis process, the membrane phosphatidylserine (PS) of the inner face of plasma membrane was externalized to the cell surface that can be easily detected by fluorescent stain Annexin V, that has a higher affinity for PS binding, then the Annexin V-PS interaction was analyzed by flow cytometry [50]. Briefly, the UO-31 cells were seeded into 12-well plate culture (2 × 10^6^ cells/well), amended with different concentrations of the extracted CPT, incubated for 48 h at standard conditions. The cells were collected and washed with phosphate buffered saline, annexin-binding buffer, followed by Annexin V-FITC and PI, according to manufacturer’s instructions. The assay was incubated in dark for 15 min at room temperature. Annexin-binding buffer was added before the flow cytometry analysis. The Annexin V-FITC binding was detected by flow cytometry (Ex, 488 nm; Em, 530 nm) with FITC signal detector and PI staining by the phycoerythrin emission signal detector.

The cell cycle of UO-31 cells was analyzed by Propidium Iodide (PI) Flow Cytometry Kit (Cat#. ab139418) according to the manufacturer’s instructions. The UO-31 cells were seeded in 12-well microtiter plate, incubated for 12 h at 37 °C, then amended with the IC_25_ value of extracted CPT, and continue incubated for 48 h. The cells were collected and fixed in 1 ml of ice-cold 70% ethanol for 2 h at 4 °C, then rehydrated with 1 ml PBS, and stained with 500 μl of PI with RNase, for 30 min at room temperature in dark. The DNA content of the cells was analyzed by the flow cytometry at Ex λ_493_ nm and Em λ_636_ nm. The percentage of G0–G1, S and G2-M cells were then calculated using Fluorescence-activated cell sorting (FACS) software.

### Bioprocessing of the CPT yield by selected fungal isolates with Plackett–Burman Design

The nutritional requirements of the potent isolates were optimized to maximize their yield of CPT with by the Plackett–Burman design [21–23, 51–53]. Nineteen variables namely, malt extract, yeast extract, glucose, sucrose, salicylic acid, asparagine, glutamine, cysteine, tryptophan, glycine, phenylalanine, peptone, pH, incubation time, sodium acetate, citric acid, CaCl_2,_ NaCl, methyljasmonate were optimized by Plackett–Burman design. The nineteen parameters assessed by Plackett–Burman design were represented by high (+ 1) and low (− 1) levels. Statistical nutritional optimization has been used frequently to evaluate the interactions of the independent factors and their consequences on the response CPT yield, unlike to the traditional optimization method (one-factor-at-time). The design of Placket-Burman depends on the first order reaction: Y = β0 + ΣβiXi.

Y is the predicted CPT production, Xi is an independent variable, βi is the linear coefficient, and β0 is the model intercept. All the runs were conducted in triplicates and the average of CPT production was used as response.

### Metabolic biosynthetic stability of CPT productivity by the potent fungal isolates

The metabolic biosynthetic stability of CPT by the potent fungal isolate was assessed with the fungal storage and subculturing. The axenic CPT-producing fungal culture was successively sub-cultured for 9 generations with the a plug centrally inoculated on PDA plate incubated at 30 °C for 8 days lifespan [25, 53, 54]. The fungal productivity for CPT was determined by growing on the optimized media, incubated at standard conditions, and then the CPT was extracted and quantified by HPLC.

As well as, the axenic 1st fungal culture was stored as slope PDA culture at 4 °C, was tested for their CPT productivity by growing on PDA media, monthly along 7 months, and the CPT was extracted and quantified as determined above.

### Restoring the biosynthetic potency of *A. terreus *CPT upon addition of organic extracts and indigenous microbiome of *C. roseus*

To restore the metabolic biosynthetic potency of CPT by *A. terreus,* different organic extracts of *C. roseus* (methylene chloride, methanol, ethylacetate, petroleum ether, and isopropyl alcohol) were amended to the CPT production medium. Ten grams of fresh leaves of *C. roseus* were pulverized in each solvent (100 ml) for 12 h, the extracts were filtered, centrifuged, and concentrated to 20 ml. The plant extracts were added to the 3 days old pre-fungal cultures at concentrations 1, 5 and 10 ml, and the cultures were incubated for 15 days under the standard conditions. After incubation, CPT was extracted and quantified by HPLC.

The influence of the indigenous microbiome of *C. roseus* leaves on restoring the biosynthetic potency of CPT by *A. terreus* was assessed. The leaves of *C. roseus* were sectioned into small parts, surface sterilized and amended into 3 days old culture of *A. terreus* grown on PDB medium, and the cultures were continue for 15 days incubation, then the CPT was extracted and quantified by HPLC. Surface sterilized leaves of *C. roseus* were inoculated into blank PDB media at the same concentrations, and used as control, regarding to the *A. terreus* culture without plant parts.

### Fungal deposition

The isolate *Aspergillus terreus* EFBL-NV was deposited into the Genbank with accession number OR131583.1.

### Statistical analysis

The experiments were conducted in triplicates, and the results were expressed by the mean ± SD. The statistical analyses were conducted by one-way ANOVA, and Tukey’s HSD test was determined by CoStat software (CoStat 2005; Version 6.311).

## Results

### Isolation of the fungal endophytes of *Catharanthus roseus*; Screening for CPT production, and Molecular identification

**Fig. 1:**
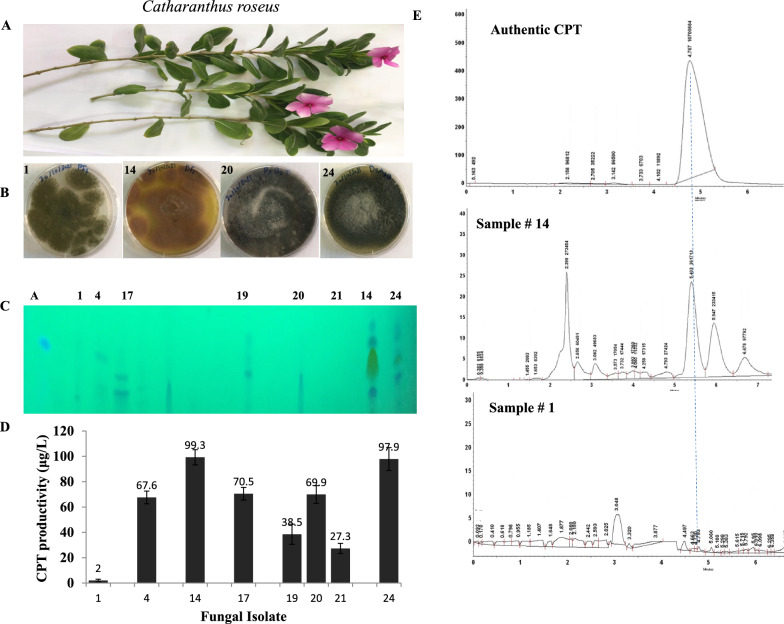
Screening for CPT production by the fungal endophytes of *Catharanthus roseus*. After incubation, CPT was extracted and screened by TLC and the yield of the most promising CPT producing isolates was quantified by HPLC. **A** Morphological view of the leaves and flowers of *C. roseus.* **B** Selected CPT producing endophytic fungal isolates. **C**, TLC profile of the recovered endophytic fungal isolates for CPT screening. Five μl of each sample were spotted to the TLC plate, compared to the authentic CPT (5 μl of at 50 μg/ml). **D** Yield of CPT from the most potent fungal isolates quantified by Image J Software Package. **E** HPLC chromatogram of the highest CPT producing isolates 14, normalized to the sample # 1, as non CPT-producers

Twenty-five fungal isolates were isolated from the twigs, leaves and flowers of *C. roseus,* these fungal isolates were morphologically identified based on their macro and microscopical features according to the universal identification keys. Ten fungal isolates were recovered on PDA medium and 15 isolates were recovered on Czapek’s-Dox medium. These fungi belong to the genera; *Aspergillus, Penicillium*, *Alternaria*, *Rhizopus* and *Trichoderma*. Practically, ten endophytic fungal isolates were recovered from the flowers, and fifteen isolates were recovered from the leaves of *C. roseus* (Additional file 1: Table S1)*.* The recovered fungal isolates was grown on PDB media, incubated at the standard conditions, CPT was extracted and quantified by TLC and HPLC. From the screening profile (Fig. 1B–D), the highest CPT productivity was reported for *Aspergillus terreus*, an endophytes of *C. roseus* flowers, (90.3 μg/L), followed by *Alternaria brasicola* (97.9 μg/l), *A. fumigatus* (69.9 μg/l), and *A. flavus* (67.6 μg/l). The yield of CPT was verified by HPLC, the HPLC chromatogram of the most potent CPT producer “*A. terreus”* in addition to non CPT producer as negative control was shown (Fig. 1D). The putative sample gave the same retention time (4.7 min) as the authentic one, ensuring its chemical proximity as CPT. The remaining endophytic fungal isolates from the flowers and leaves of *C. roseus* lacks the metabolic potency to produce CPT. Interestingly, the most potent CPT producer *A. terreus* NV1 were recovered from the flowers, however, three isolates of *A. terreus* NV2, NV3, and NV4 were recovered from the leaves of *C. roseus* with tiny yield of CPT, suggesting the dependence of expression of CPT biosynthetic genes on microbiome of plant flower than the leaves. The obvious fluctuation on the yield of flowers inhabited *A. terreus* NV1 and other leaves inhabited *A. terreus* isolates, ensures the key role of the fungal-plant host interaction, host physiological and biochemical identities on modulating the selective expression of CPT encoding genes.

**Fig. 2:**
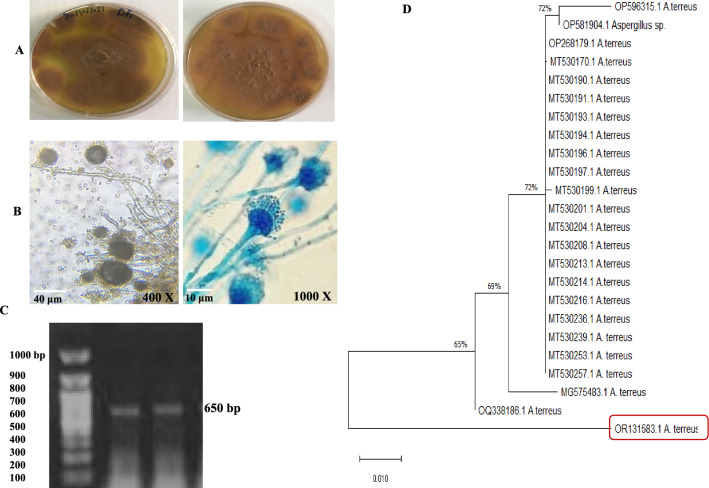
Morphological and molecular identification of *A. terreus* as the most potent CPT producer. **A** Plate culture of *A. terreus* on PDB after 6 days of incubation at 30 °C. **B** The microscopical view of the conidial heads of *A. terreus* at 400 × (scale bar 40 μm), and 1000 × (scale bar 10 μm). **C** PCR amplicon of the ITS regions of *A. terreus,* normalized to the DNA ladder (1 kb Nex-gene Ladder, Puregene, Cat.# PG010-55DI). **D** Molecular phylogenetic analysis of *A. terreus*, an endophyte of *C. roseus* by Maximum Likelihood method

The morphologically identified potent CPT-producing endophyte of *C. roseus* “*A. terreus* NV1” was molecularly confirmed based on the sequence of ITS region. The amplicon of the ITS region of the fungal isolate was ~ 650 bp (Fig. 2). The PCR amplicon was sequenced, and ITS sequence was non-redundantly BLAST searched on the NCBI database. The ITS sequence of *A. terreus* EFBL-NV1 was deposited to the Genbank with accession number OR131583.1. From the alignment profile and phylogenetic analysis of ITS sequences, the isolate *A. terreus* EFBL-NV displayed 99% similarity with various *A. terreus* isolates of accession # MG575483.1, MT530257.1, MT530236.1, MT530216.1, MT530214.1, MT530208.1, MT530201.1, MT530199.1, MT530197.1, MT530197.1, MT530196.1, MT530194.1, MT530193.1 and MT530191.1 with E-value zero and 99% query coverage.

### Chromatographic, spectroscopic analyses, and LC–MS/MS analyses of the extracted CPT

**Fig. 3:**
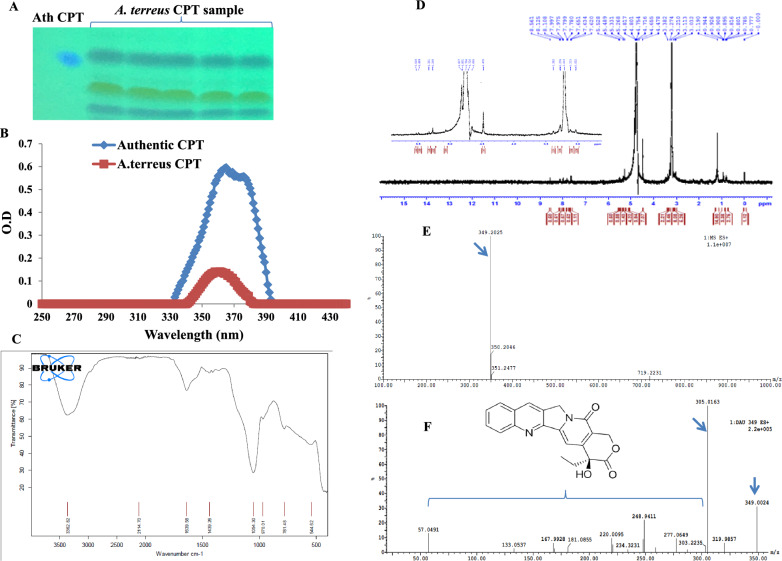
Chromatographic and spectroscopic analysis of the purified CPT of *A. terreus*. **A** TLC chromatogram of the putative CPT, the target spots were scraped-off from the plates and used for further analyses. **B** UV-spectra of the purified CPT, compared to the authentic one. **C** FT-IR spectra of the putative CPT and authentic one. **D** The HNMR spectra of the putative CPT of *A. terreus*. **E** The LC–MS analysis of the putative CPT of 349 m/z. **F** MS/MS fragmentations of the parent CPT molecule (349 m/z). Ath, refers to authentic CPT

The identity of the putative CPT from *A. terreus* was confirmed by the UV–Vis, FTIR, HNMR, and LC–MS/MS analyses, compared to the authentic CPT. After cultural incubation, CPT was extracted, fractionated by TLC, and the spots of CPT containing silica gel with the same mobility and color, were scraped-off and dissolved in methanol for chemical analysis (Fig. 3A). The purified CPT from *A. terreus* had the same VU-absorption pattern of the authentic CPT, with maximum absorbance at wavelength 360 nm (Fig. 3B). From the FTIR spectra, the purified CPT of *A. terreus* had a peak at 3406.6 and 3393.3 cm^−1^ that were assigned for the hydroxyl (OH) and amide group stretches, respectively. As well as, the CPT had a distinct peak of 2923.5, 1729.8 and 1604.5 cm^−1^ that was assigned to the aliphatic CH, ester groups and aromatic rings stretch, respectively. The COO stretching frequency peaks at 1268.9 cm^−1^, 1029.8 cm^−1^ were assigned for the aromatic C and H blends. The distinct peaks of CPT was resolved at 3438, 1666, 1113 and 1035 cm^−1^ that refers to the stretching of OH, C=O, C=N, C–C(=O)–O and C–O functional groups, respectively (Fig. 3C). From the FTIR spectrum, the purified CPT from *A. terreus* had the same functional groups orientation and stretching patterns of authentic one, ensuring the chemical identity of the purified sample as CPT. The chemical structure of the CPT from *A. terreus* was resolved from the HNMR displayed the same signals of the authentic one, distributed between 1.0 and 8.0 ppm, with three proton signals resolved at 1.0–2.5 ppm corresponding to methyl, acetate and acetylene groups, and signals for aromatic moieties resolved at 7.0–8.4 ppm (Fig. 3).

The molecular identity of CPT has been confirmed by the LC–MS/MS analysis at positive mode. The CPT of *A. terreus* had the same molecular mass to charge ratio (349.2 m/z) of the authentic CPT from *Camptotheca acuminata* [1]. Moreover, the parent CPT molecule (349.2 m/z) was further fragmented by MS/MS applying collision energy of 35 electron Volts (eV), the fragments of molecular mass 57.0, 133.1,167.9, 181.08, 220.009, 234.32, 248.94, 277.06, 303.2 and 305.01 m/z were recovered, with the same fragmentation pattern of the authentic CPT. From the 1st mass spectra, a peak at retention time 5.67 min with a molecular ion peak at m/z 349.12 [M + H] + corresponding to the molecular formula C_20_H_16_N_2_O_4_. The peaks at retention times 5.67, 5.76, 5.84 min, exhibited a protonated molecular ion peak [M + H] + of CPT at m/z 349.12. Thus, the putative sample of *A. terreus* has been chemically authenticated as CPT, normalizing to the authentic one.

### Guided-activity of the putative CPT against the CPT producing and non-producing fungi

**Fig. 4:**
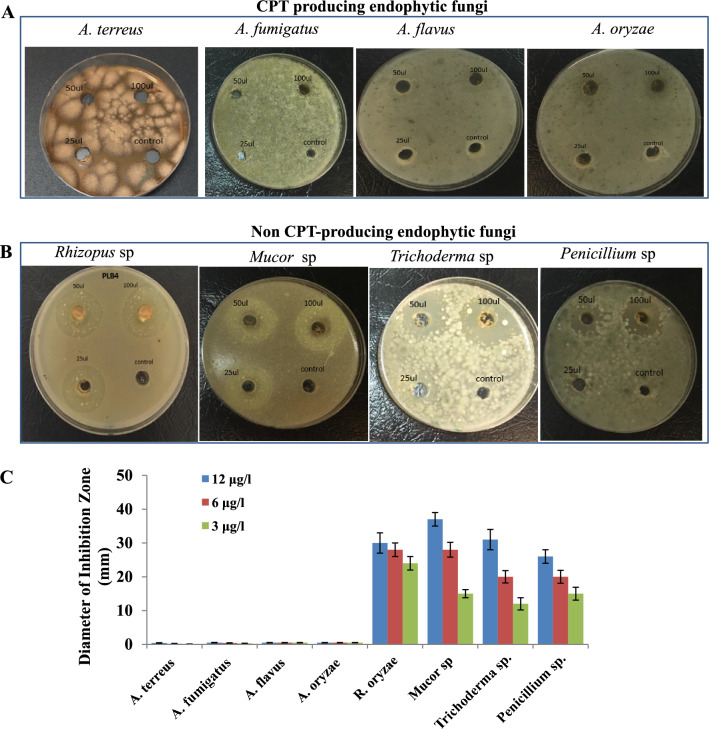
Antimicrobial activity of the extracted *A. terreus* CPT towards CPT producing and non-producing fungi. The CPT spots were scrapped-off from the TLC silica gel plates, eluted, and different concentrations of the CPT (5, 10 and 15 μg/ml) was applied to the tested fungal cultures, incubated for 5 days, then the diameter of the inhibition zone was measured. After **A** The panel of CPT-producing fungi (*A. terreus, A. fumigatus, A. flavus, A. oryzae*). **B** The panel of non CPT-producing fungi (*Rhizopus* sp, *Mucor* sp, *Trichoderma* sp, and *Penicillium* sp). **C** The diameter of the inhibition zone of the CPT producing and non CPT-producing fungi by the purified *A. terreus* CPT

The antimicrobial activity of the metabolites has been used frequently as preliminary signs of antiproliferative, since the physiological behaviors of the microbial cells are mostly similar to tumor cells. The activity of the extracted CPT from *A. terreus* was assessed against the CPT producing fungi, as well as, against the non-producing fungal endophytes of *C. roseus*. The CPT of *A. terreus* was purified, and assessed towards the positive CPT producing fungi; *A. terreus*, *A. fumigatus* and *A. flavus* and *A. oryzae*, in addition to the non-producing isolates; *Rhizopus oryzae*, *Mucor *sp, *T. atrovirdie* and *P. polonicum*. From the results (Fig. 4), the extracted CPT from *A. terreus* NV1 had no activity towards the CPT producing fungal isolates, in contrary to the dramatic activity towards the non-CPT producing fungi. Obviously, the activity of *A. terreus* CPT towards the non-CPT producing fungi has been observed as a concentration-dependent manner as revealed from the inhibition zones (Fig. 4B, C). The diameter of inhibition zone of the *A. terreus* CPT for *Rhizopus oryzae*, *Mucor *sp*, T. atroviride* and *P. polonicum* were ranged between 27 and 37 mm in response to CPT concentration 12 μg/ml, normalized to 10% DMSO as a negative control*.* Interestingly, the lack of effect of CPT on CPT-producing fungi, in contrary to the strong inhibitory effect on the non- producing fungi, ensures the possessing of former fungi to a specific mechanism of resistance to CPT effect, that might be by blocking the transportation of this compound to the cytosol of fungal cells or altering the orientation of topoisomerases I, II targets to be inaccessible for CPT binding.

### Antiproliferative, topoisomerases inhibition and wound healing activity of the extracted *A. terreus* CPT

**Fig. 5:**
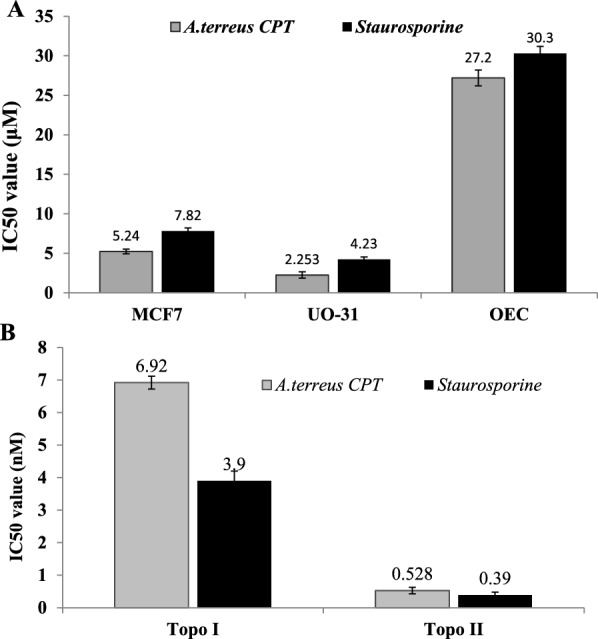
Antiproliferative activity and kinetics of inhibition of Topoisomerase I and II by the purified CPT from *A. terreus.* **A** The IC50 values of the purified *A. terreus* CPT towards the MCF7 and UO-31 cell lines, compared to the normal OEC. **B** Kinetics of inhibition of topoisomerase I and II by CPT of *A. terreus*, compared to Staurosporine as reference drug

The activity of the extracted *A. terreus* CPT was assessed against MCF7 and UO-31 cell lines, at different CPT concentrations (1–10 μM). From the calculated IC_50_ values (Fig. 5A), the extracted *A. terreus* CPT had a significant activity towards the MCF-7 (5.2 μM) and UO-31 (2.25 μM) cell lines, compared to staurosporine as authentic anticancer drug, that has 7.8 μM and 4.2 μM, towards the cell lines, respectively. So, the extracted CPT of *A. terreus* displayed a powerful activity towards the MCF7 and UO-31, than the authentic anticancer drug “staurosporine”. From the IC_50_ values, the activity of *A. terreus* CPT towards UO-31 was ~ two-folds higher than MCF7, ensuring the susceptibility of UO-31 to CPT, that might due to feasibility of entrance to the cytosol and binding with topoisomerases. The higher sensitivity of UO-31 to CPT, might be related to the structural activity relationships of binding the CPT with the topoisomerases, in addition to targeting another metabolic process and/or structural organelles.

The ability of the purified *A. terreus* CPT to inhibit the DNA topoisomerases 1 and II was assessed. Different CPT concentrations were amended to the reaction assay of Topo I, II, and the residual enzymatic activity was determined. From the results (Fig. 5B), the purified *A. terreus* CPT displayed a significant activity towards topoisomerase II than I by about 13 folds, that being matched with the results of staurosporine. The IC_50_ value of the *A. terreus* CPT towards topoisomerase I and II was 6.9 nM and 0.52 nM, respectively. The inhibitory effect of *A. terreus* CPT and staurosporine was closely similar for the topoisomerase II, however, the CPT displayed a higher binding affinity for topoisomerase I by about two-folds, that might related to the structural activity relationship (SAR) of the CPT and staurosporine for binding with topoisomerase I.

**Fig. 6:**
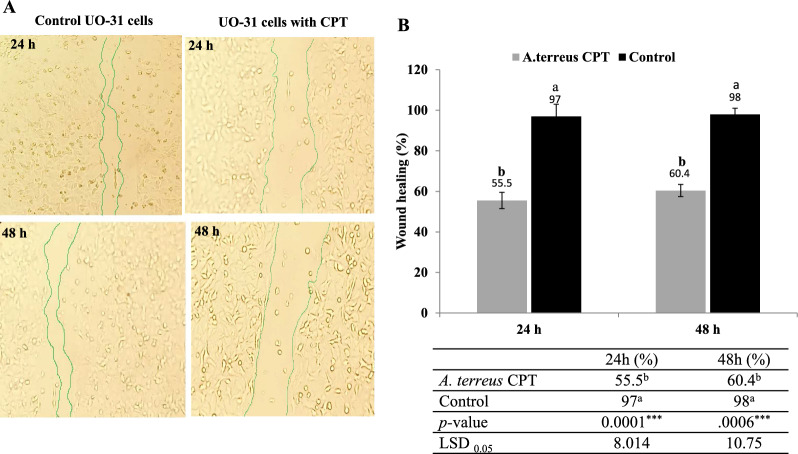
Wound healing assay of the UO-31 cells in response to *A. terreus* CPT after 24 and 48 h comparing to the untreated cell lines (control). After 24 h of growth of UO-31 cells as homogenous monolayer, a scratch was made and the tested CPT was added to the well at final concentration 0.25 μM. **A** Wound healing of UO-31 cells in response to *A. terreus* CPT after zero time, 24 and 48 h. **B** The percentage of wound healing of the UO-31 cells in response to *A. terreus* CPT. The statistical analysis results of the one-way ANOVA was summarized. The values were represented by the means, followed by letters a, b within the same column that is a significantly different (ONE Way ANOVA, LSD test, p ≤ 0.05). ns refers non-significant, *refers to significant difference, **refers to highly significant difference. LSD is the least significant difference

The wound healing activity of UO-31 in response to *A. terreus* CPT treatment was assessed, by inspecting the gap closure after 24 and 48 h, comparing to untreated cells (control). The UO-31 cells were used for further cell cycle and apoptosis analyses, due to their sensitivity to the purified CPT (IC50 value 2.5 μM) compared to MCF-7 cell (IC50 value 5.2 μM). Obviously, the percentage of scratch/gap closure was noticeably inhibited upon treatment with *A. terreus* CPT, with the incubation time, compared to the control cells (Fig. 6A). Practically, the wound healing of the homogenous monolayer of UO-31 cells was approximated by about 55.5% compared to 97% of control cells, after 24 h (Fig. 6B). With the prolongation of incubation time to 48 h, the wound closure of UO-31 cells was recorded by about 98% and 60.4% for the untreated and CPT treated cells, respectively. The remarkable wound healing suppression ensures the interference of CPT with the cell regeneration, and matrix formation of the UO-31 tumor cells. So, the dual activity of CPT by binding with topoisomerases I and II, in addition to prevent the cellular matrix formation and motility seems to be more therapeutically affordable.

### Apoptosis and cell cycle analysis of UO-31 in response to CPT of *A. terreus*

**Fig. 7:**
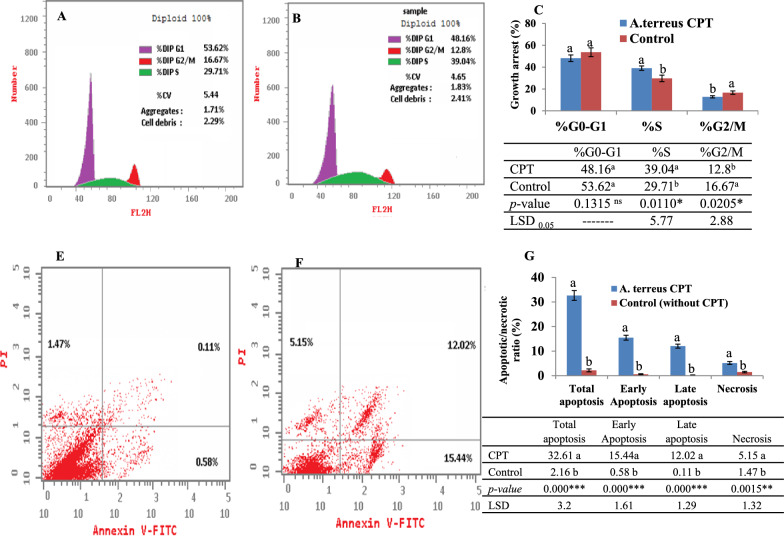
Cell cycle and apoptosis of UO-31 in response to CPT of *A. terreus.* The cell cycle of UO-31 cells without CPT (**A**), and treated with *A. terreus* CPT (**B**), and the overall cellular growth arrest (**C**) in response CPT compared to control. Cell cycle analysis by Annexin-V-PI of UO-31 cells without CPT (**E**), with *A. terreus* CPT (**F**) and the overall apoptotic ratios (**G**). The statistical analysis results of the one-way ANOVA was summarized. The values were represented by the means, followed by letters a, and b within the same column that is a significantly different (ONE Way ANOVA, LSD test, p ≤ 0.05). ns refers non-significant, *refers to significant difference, **refers to highly significant difference. LSD is the least significant difference

The apoptotic process of UO-31 in response to the CPT of *A. terreus* was assessed by Annexin V-PI assay that mainly based on the externalization of membrane phosphatidylserine (PS) in early stages of apoptosis, forming Annexin V-PS complex that can be easily analyzed by flow cytometry, to elucidate the different apoptosis stages. From the flow cytometry results (Fig. 7A–C), a significant shift of the normal cells to apoptotic phase was observed in response to the CPT of *A. terreus*, compared to control cells (untreated cells). Upon treatment with *A. terreus* CPT, the percentage of the UO-31 cells in early apoptosis, late apoptosis, and necrosis were ~ 15.5%, 12.02% and 5.1%, respectively. However, the percentage of early apoptosis, late apoptosis, and necrosis were 0.58%, 0.1% and 0.47%, respectively. So, upon addition of *A. terreus* CPT, the total apoptosis of UO-31 cells was increased by about 16 folds, compared to the untreated cells.

The cell cycle of UO-31 was analyzed in response to addition of CPT of *A. terreus,* by propidium iodide assay. The cells were amended with the IC_25_ values (1.1 μM) of CPT, incubated, collected and fixed in ice-cold ethanol, and the percentage of G0-G1, S and G2-M cells were calculated. From the cell cycle analysis (Fig. 7E–G), the growth of UO-31 cells was maximally arrested at S-phase, compared to the control cells without treatments. However, a similar effect has been observed for UO-31 cell at the G0-G1 and G2-M phases for the treated and untreated cells. Overall, CPT of *A. terreus* had a noticeable inhibitory effect to the cells at the S-phase, as revealed from the maximum growth arrest, compared to the other cell cycle phases.

### Bioprocess of CPT production by *A. terreus* using the Plackett–Burman design

**Fig. 8:**
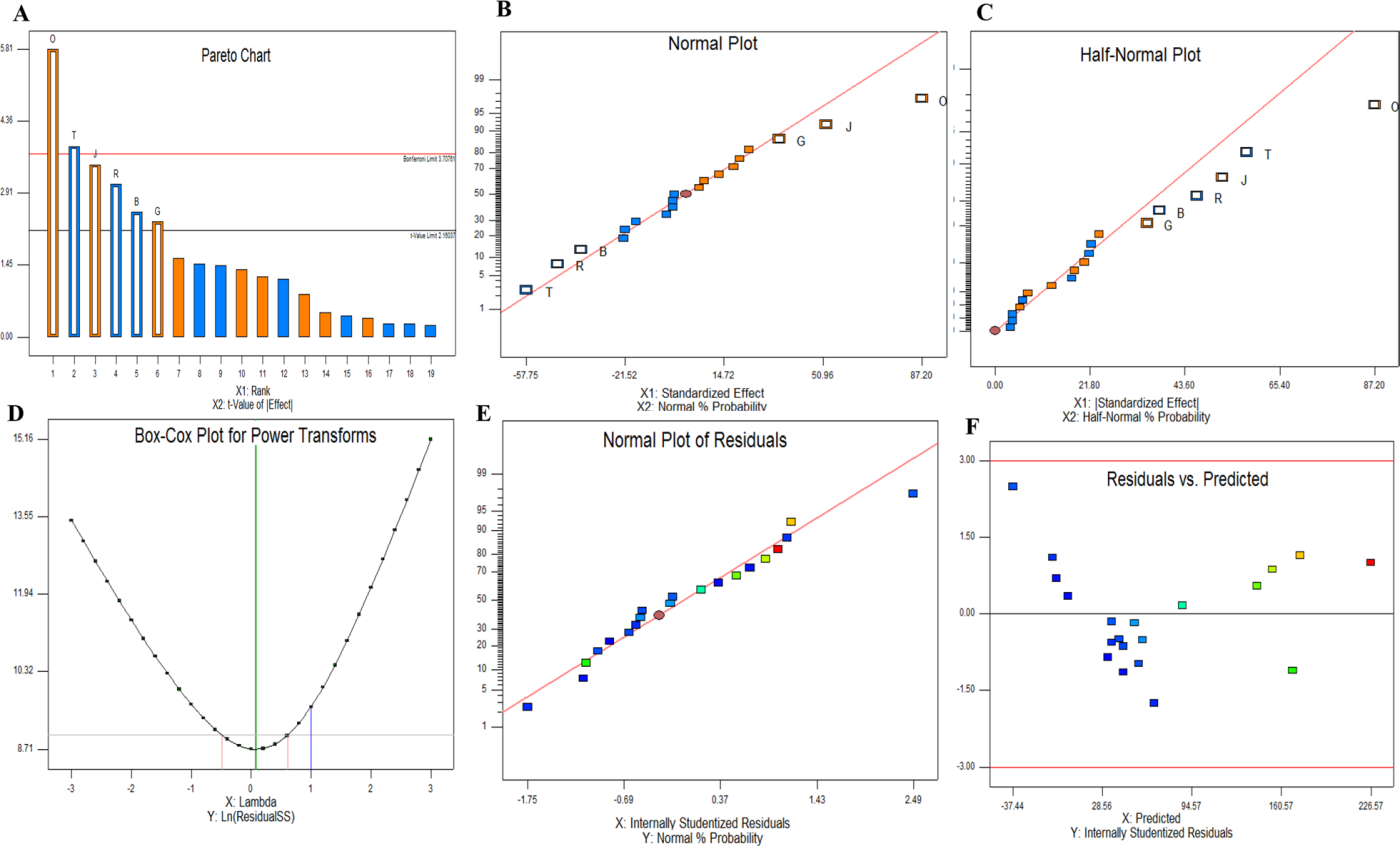
The main effects of different variables on CPT production by *A. terreus* with the Plackett–Burman experimental design. **A** Pareto chart illustrates the order of significance of each variable. Normal plot (**B**) and half-normal (**C**) of probability with standardized effect. **D** Box-Cox of power transform. **E** Normal plot of the internally standardized residuals. **F** Plots of residuals versus predicted response of CPT by *A. terreus* Plot of the correlation of the predicted and actual camptothecin yield by *A. flavus*. **E** Normal plot of the residual

**Fig. 9:**
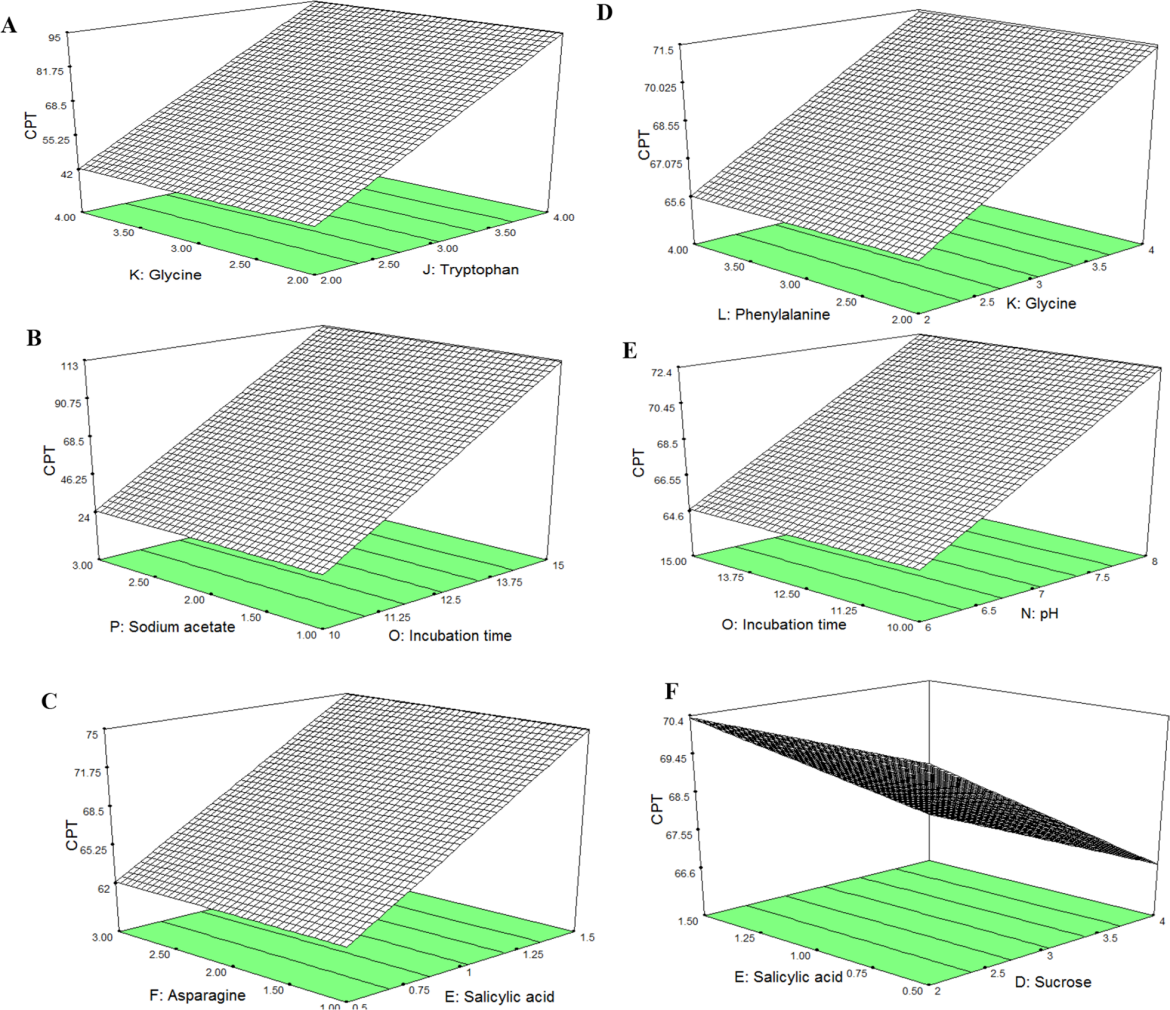
Three-dimensional surface plots for interactions of the variables for CPT production. The interaction of for glycine and tryptophan (**A**), sodium acetate and incubation time (**B**), asparagine and salicylic acid (**C**), phenylalanine and glycine (**D**), incubation time and pH (**E**), and salicylic acid and sucrose (**F**)

The productivity of CPT by *A. terreus* was maximized by the nutritional optimization, since the chemical components and their interactions are essential in controlling the biosynthesis of bioactive secondary metabolites [21–24, 33, 51]. The nutritional requirements for maximum CPT production by *A. terreus* were optimized by Plackett–Burman design as 1st order model. The tested nineteen parameters including the various carbon, nitrogen, growth elicitors, growth modulators and physical factors for growth of *A. terreus* were studied with their lower and higher values (Table 1). The impact of the tested variables affecting CPT productivity by *A. terreus*, with the predicted, corresponding actual responses, and their residuals were summarized in Table 2. The actual and predicted yield of CPT by *A. terreus* were noticeably fluctuated from 9.3 to 255.5 μg/L confirming the significance of tested variables on CPT biosynthesis, reveals the efficiency of the Plackett- Burman design. The *F*-value (9.8), *p*-value (< 0.0007) and adjusted determination coefficient (Adj. R^2^ = 0.92) refers to the efficiency of the model as shown in Table 3. The main effects, normal probability of the tested factors were plotted (Fig. 8), revealing the six different independent factors including the incubation time, yeast extract, glutamine, tryptophan, CaCl_2_ and methyljasmonate that have a significant effect on CPT productivity by *A. terreus.* The 3D surface response methodology plots of the most significant variables affecting CPT productivity of *A. terreus* was illustrated in Fig. 9 The maximum yield of CPT (255.6 μg/l) by *A. terreus* was reported at run# 10, with the medium components malt extract (+ 1), yeast extract (− 1), glucose (+ 1), sucrose (− 1), salicylic acid (− 1), asparagine (+ 1), glutamine (+ 1), cysteine (− 1), tryptophan (+ 1), glycine (+ 1), phenylalanine (− 1), peptone (− 1), pH (+ 1), incubation time (+ 1), sodium acetate (+ 1), citric acid (+ 1), CaCl_2_ (− 1), NaCl (+ 1) and methyljasmonate (− 1). The lowest CPT yield (9.1 μg/l) was recorded at run # 19 and run # 4, respectively. From the ANOVA analyses, the model was highly significant as reveled from the values of Fisher’s f-test 13.6 and probability *p*-value 0.0001. From the Plackett–Burman design, the most significant variables affecting CPT productivity by *A. terreus* was the yeast extract, glutamine, tryptophan, incubation time, CaCl_2_, and methyljasmonate. The actual yield of *A. terreus* CPT was fluctuated from 255.1 to 9.1 μg/l, confirm the significance of the tested variables on biosynthesis of CPT. So, the optimal components for the maximum CPT production by *A. terreus* contains Yeast Extract (− 1), Glutamine (+ 1), Tryptophan (+ 1), Methyl jasmonate (− 1), and CaCl_2_ (− 1), after 15 days of incubation time. The first order polynomial equation for camptothecin produced by *A. terreus* regarding to the significant independent variables was derived from the following equation,

$$\begin{aligned} {\text{CPT}}\, = \, & - {78}.{94547 } - {18}.{873}0{8 }*{\text{ Yeast Extract 17}}.{49144 }*{\text{ Glutamine26}}.{1}0{1}0{1}*{\text{ Tryptophan 17}}.{439}0{7 }*{\text{ Incubation time }} \\ & - {92}.{657}0{8 }*{\text{ CaCl2}} - {144}.{37964 }*{\text{ Methyl jasmonate}} \\ \end{aligned}$$

**Table 1:** The coded and actual values for the tested variables

Codes	Factors	Levels	
		− 1	1
X1	Malt extract	2	4
X2	Yeast extract	2	4
X3	Glucose	4	6
X4	Sucrose	2	4
X5	Salicylic acid	0.5	1.5
X6	Asparagine	1	3
X7	Glutamine	1	3
X8	Cysteine	1	3
X9	Tryptophan	2	4
X10	Glycine	2	4
X11	Phenylalanine	2	4
X12	Peptone	2	5
X13	pH	5	8
X14	Incubation time	10	15
X15	Sodium acetate	1	3
X16	Citric acid	1	3
X17	CaCl2	0.5	1.0
X18	NaCl	0.5	1.0
X19	Methyljasmonate	0.2	0.6

**Table 2:** Matrix of the Plackett–Burman Design for optimization of CPT production from *A. terreus*

Std. order	X1	X2	X3	X4	X5	X6	X7	X8	X9	X10	X11	X12	X13	X14	X15	X16	X17	X18	X19	CPT yield (µg/l)	Predicted (µg/l)
1	− 1	− 1	− 1	− 1	1	1	− 1	1	1	− 1	− 1	1	1	1	1	− 1	1	− 1	1	91.8	87.5
2	− 1	1	1	− 1	1	1	− 1	− 1	1	1	1	1	− 1	1	− 1	1	− 1	− 1	− 1	177.3	153.8
3	1	1	1	− 1	1	− 1	1	− 1	− 1	− 1	− 1	1	1	− 1	1	1	− 1	1	1	21.5	3
4	− 1	1	1	− 1	− 1	1	1	1	1	− 1	1	− 1	1	− 1	− 1	− 1	− 1	1	1	13.1	43.8
5	1	− 1	1	1	− 1	− 1	1	1	1	1	− 1	1	− 1	1	− 1	− 1	− 1	− 1	1	138.8	168.8
6	1	− 1	1	− 1	− 1	− 1	− 1	1	1	− 1	1	1	− 1	− 1	1	1	1	1	− 1	44.27	58.1
7	− 1	1	1	1	1	− 1	1	− 1	1	− 1	− 1	− 1	− 1	1	1	− 1	1	1	− 1	157.3	142.4
8	1	1	− 1	− 1	1	1	1	1	− 1	1	− 1	1	− 1	− 1	− 1	− 1	1	1	− 1	12.4	3.1
9	− 1	− 1	− 1	1	1	− 1	1	1	− 1	− 1	1	1	1	1	− 1	1	− 1	1	− 1	205.4	174.3
10	1	− 1	− 1	− 1	− 1	1	1	− 1	1	1	− 1	− 1	1	1	1	1	− 1	1	− 1	255.1	226.5
11	− 1	− 1	− 1	− 1	− 1	− 1	− 1	− 1	− 1	− 1	− 1	− 1	− 1	− 1	− 1	− 1	− 1	− 1	− 1	47.3	52.2
12	1	1	− 1	1	− 1	1	− 1	− 1	− 1	− 1	1	1	− 1	1	1	− 1	− 1	1	1	26.7	43.8
13	1	− 1	1	− 1	1	− 1	− 1	− 1	− 1	1	1	− 1	1	1	− 1	− 1	1	1	1	31.1	35.3
14	− 1	1	− 1	1	− 1	− 1	− 1	− 1	1	1	− 1	1	1	− 1	− 1	1	1	1	1	29.9	18
15	1	1	− 1	1	1	− 1	− 1	1	1	1	1	− 1	1	− 1	1	− 1	− 1	− 1	− 1	19.2	66.6
16	− 1	− 1	1	1	− 1	1	1	− 1	− 1	1	1	1	1	− 1	1	− 1	1	− 1	− 1	27.5	12
17	1	− 1	− 1	1	1	1	1	− 1	1	− 1	1	− 1	− 1	− 1	− 1	1	1	− 1	1	20.1	35.2
18	− 1	− 1	1	1	1	1	− 1	1	− 1	1	− 1	− 1	− 1	− 1	1	1	− 1	1	1	13.2	17
19	− 1	1	− 1	− 1	− 1	− 1	1	1	− 1	1	1	− 1	− 1	1	1	1	1	− 1	1	9.6	32.5
20	1	1	1	1	− 1	1	− 1	1	− 1	− 1	− 1	− 1	1	1	− 1	1	1	− 1	− 1	28.8	55.3

**Table 3:** ANOVA for selected factorial model, Analysis of variance table [Partial sum of squares—Type III]

Source	Sum of squares	df	Mean square	F-value	Prob > F	
Model	92,291.32	6	15,381.89	13.67	< 0.0001	Significant
B-Yeast extract	7123.86	1	7123.86	6.33	0.0258	
G-Glutamine	6119.01	1	6119.01	5.44	0.0364	
J-Tryptophan	13,625.26	1	13,625.26	12.11	0.0041	
O-Incubation time	38,015.14	1	38,015.14	33.79	< 0.0001	
R-CaCl_2_	10,731.67	1	10,731.67	9.54	0.0086	
T-Methyl jasmonate	16,676.38	1	16,676.38	14.82	0.002	
Residual	14,625.95	13	1125.07			
Cor total	1.07E + 05	19

So, upon Placket-Burman optimization process, the yield of CPT by A. terreus was increased by about 2.8 folds (255 μg/l) compared to the control PDB medium (~ 90.1 μg/l).

### Productivity of CPT by *A. terreus* with the subculturing and storage

**Fig. 10:**
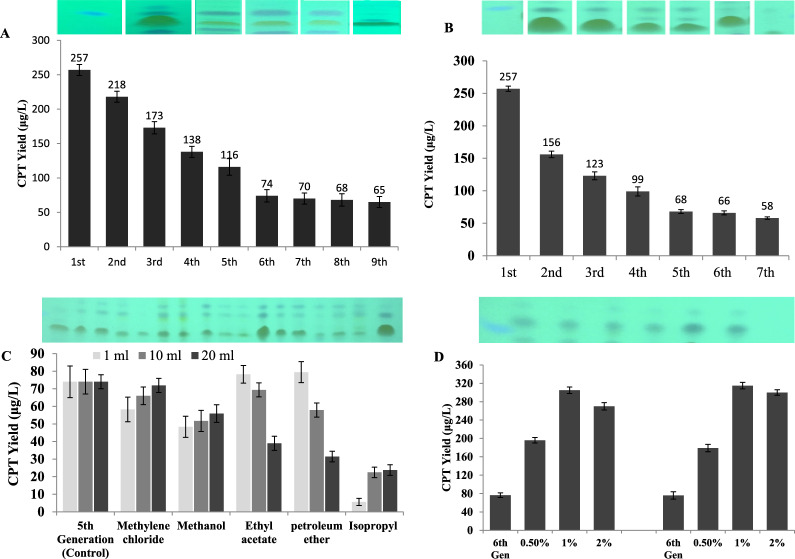
Metabolic stability of *A. terreus* for CPT production with the fungal subculturing and storage. The fungal isolate was grown on PDB for 8 days, and CPT was extracted and quantified. The yield of CPT of *A. terreus* in response to fungal subculturing (**A**), and storage for 7 months (**B**). The upper panels are the TLC and lower panels were the yield quantified by Image J. **C**, The yield of CPT by the 5th generation of *A. terreus* amended with different organic solvents extracts of *C. roseus.* **D**, The yield of CPT of 5th culture of *A. terreus* amended with surface sterilized leaves and flowers of *C. roseus*

The biosynthetic stability of *A. terreus* for CPT production with the subculturing and storage was assessed. The 1st isolate of *A. terreus* preserved as slant cultures on PDA for 8 days at 30 °C, was subcultured till the 9th generation, and their CPT productivity was determined by TLC. Practically, a noticeable loss has been observed on the CPT productivity by *A. terreus* with the successive subculturing (Fig. 10A,). The yield of CPT by the 1st culture of *A. terreus* (257 μg/l) was reduced by ~ 2.2 folds by the 5th generation (116 μg/l). At the 7th subcultures, the yield of CPT by *A. terreus* was reduced by 3.7 folds (70 μg/l), compared to the 1st culture. So, attenuation of the yield of CPT with the successive subculturing of *A. terreus* has been noticeably recorded.

In addition, the effect of storage of *A. terreus* as slope culture on PDA at 4 °C has been evaluated, intervally till 7th months. Remarkably, *A. terreus* lost their productivity of CPT by about 50%, by the 3rd month of storage as slope culture at 4 °C. The CPT yield of the 1st *A. terreus* culture (257.2 μg/l) was decreased into 68.1 μg/l by storage by the 5th month, with ~ 3.7 folds reduction (Fig. 10B).

### Effect of organic solvent extracts and indigenous microbiome of *C. roseus* on restoring the CPT productivity of *A. terreus*

Reduction of productivity of CPT by fungi with their subcultures and storage is the major metabolic change that limits the further industrial applications of fungi [21–24, 33]. Several hypotheses unravel the fungal-plant interactions, revealing the dependence of the biosynthetic machinery of CPT by the endophytic fungus on some chemical signals from the plant or from their indigenous microbiome. So, the 5th *A. terreus* culture was amended with different organic solvents extracts of *C. roseus*, incubated at standard conditions, then the CPT was extracted and quantified by HTLC and PLC. From the results (Fig. 10C), the organic solvent extracts of *C. roseus* “methanol, dichloromethane, ethylacetate, petroleum ether, isopropyl alcohol” had no an obvious effect on restoring the biosynthesis of CPT by *A. terreus*. The negative effect of the utilized wide-range polarity solvents on the yield of CPT negates the association of prompting signals from the host plants, or weakening of these signals during the downstream extraction processing.

The biosynthetic potency of CPT by the 5th *A. terreus* culture was assessed in response to addition of different plant parts. Interestingly, the yield of CPT by *A. terreus* was strongly restored and enhanced upon addition of surface sterilized parts of *C. roseus* flowers and plant twigs. The plant parts without *A. terreus* were used a negative controls. The yield of CPT by the 5th culture of *A. terreus* was maximally increased by addition of 1% of *C. roseus* flowers (305 μg/l) and twigs (315 μg/l). So, the overall yield of CPT by 5th *A. terreus* was completely restored and increased over the 1st culture of *A. terreus* by about by 1.3 folds, upon addition of 1% leaves of *C. roseus*. Thus, with addition of the indigenous *C. roseus* microbiome, the biosynthetic machinery of *A. terreus* CPT was reinstated.

## Discussion

CPT derivatives have been recognized as one of the most prescribed anticancer drugs for most of the solid tumors, due to their unique ability to bind with topoisomerase I of tumor cells, thus, keeping the DNA-supercoiling, and preventing the relaxation of DNA, and leading to cell death. [4]. The biosynthetic potency of CPT by endophytic fungi raise the hope for the commercial production of this compound, for the fast fungal growth, accessibility for bulk biomass, independence on environmental conditions, and feasibility of metabolic engineering, however, the anticipation of fungi for commercial production of CPT has been challenged by the loss of CPT productivity with the storage and subculturing [14, 22, 23, 29, 55]. Thus, screening for a novel fungal endophyte with higher productivity and affordable biosynthetic CPT stability was the objective. So, we have been motivated for screening of CPT production from fungal endophytes inhabiting the medicinal plants with traditional pharmaceutical uses, especially *Catharanthus roseus*. *Catharanthus roseus* is one of the most crucial world-wide medicinal plants, possessing a wide-range of phytochemicals with diverse biological activities; antioxidant, antimicrobial, and anticancer properties [56, 57]. Vinblastine and vincristine as common anticancer drug were isolated from *C. roseus* [58].

Among the recovered endophytic fungal isolates inhabiting the flowers of *C. roseus*, *A. terreus* EFBL-NV1 was recognized as the most CTP producing isolate (~ 90.1 μg/L), it was molecularly confirmed based on the ITS sequence, and deposited on Genbank with accession # OR131583.1. Consistently, isolates of *A. terreus*, endophytes of *F. elastica*, *Cestrum parqui*, *Astragalus fruticosus* and *Cinnamomum camphora* were recognized as CPT producers, ensuring the harboring of the distinct CPT biosynthetic machinery of *A. terreus* regardless to the different plant hosts [21–25, 59]. Remarkably, the common presence of isolates of *A. terreus* with potency for CPT production among various medicinal plants, declares the efficacy of the biosynthetic machinery of CPT by *A. terreus*, as reciprocal mechanism for plant protection via the fungal-plant interaction. The disparity on the yield of CPT by the isolates of *A. terreus* inhabiting different plant hosts, might be attributed to the fungal-microbiome interactions, modulating the molecular expression of the CPT encoding genes by *A. terreus* [21–24].

The chemical identity of the putative CPT from *A. terreus* was confirmed by the UV–Vis, FTIR, H NMR and LC–MS/MS analyses, ensuring the chemical identity of the purified sample as CPT. The putative CPT had the same molecular mass (349 m/z) and molecular fragmentation pattern as revealed from the MS and MS/MS, that identical to the authentic CPT of *C. accuminata* [1, 22]. The MS/MS fragmentation pattern of the current CPT sample was coincident to the fragmentation pattern of *Nothapodytes nimmoniana* [55, 60], and *A. terreus* [21, 23, 24, 59]. Thus, from the FT-IR, HNMR, LC–MS/MS, the putative sample of *A. terreus* has been chemically authenticated as CPT, normalizing to the authentic one.

The antimicrobial activity of metabolites has been used as preliminary signs for antiproliferative activity, since numerous physiological features of microbial cells are mostly identical with tumor cells. The extracted CPT had no activity against *A. terreus*, *A fumigatus*, *A. flavus* and *P. chrysogenum* “CPT producers”, unlike to the dramatic activity against *Rhizopus* sp, *Mucor* sp, *T. atroviride* and *P. polonicum,* as non CPT-producers, in a concentration-dependent manner. The lack of inhibitory effect of CPT on CPT-producing fungi, ensures the possessing of specific mechanisms of resistance to CPT effect, or by blocking the transportation of this compound to the cytosol of fungal cells, or altering the orientation of topoisomerases target to be inaccessible for CPT binding [22]. The antiproliferative activity of the extracted *A. terreus* CPT was assessed towards MCF7 and UO-31 cell lines. The extracted *A. terreus* CPT had a significant activity for the MCF-7 (5.2 μM) and UO-31 cells (2.24 μM), compared to staurosporine as authentic drug. Consistently, the antiproliferative activity of the extracted *A. terreus* CPT was coincident with CPT from various endophytic fungi [21, 22], towards various cell lines. The affinity of purified *A. terreus* CPT to inhibit the DNA topoisomerases 1 and II, was assessed. The purified *A. terreus* CPT displayed a significant activity towards topoisomerase II than I by about 13 folds. The higher affinity of *A. terreus* CPT for binding with topoisomerase II and I could be an affordable therapeutic criterion, since topoisomerase II catalyzes cleavage of both DNA strands, and down-regulation of the Topo I could be an adaptive mechanism of tumor cells to resist the CPT effect [5, 7]. The topoisomerase II is able to catalyze the relaxation of both positively and negatively supercoiled DNA. The unique affinity of *A. terreus* CPT to inhibits Topo II than Topo I, could be due to their specific structural activity relationships (SAR), of stereo-structural conformation of the current CPT, so, further molecular modeling are needed to explore the higher affinity of *A. terreus* CPT to Topo II than I. Consistently, evodiamine, a natural product from *C. acuminata* has a dual catalytic topo I/II inhibitor, exhibits an enhanced inhibition against CPT [61]. Therefore, targeting both Topo I and II simultaneously should lower the potential for the development of resistance against such inhibitors [6]. Human Topo II is an effective target in the treatment of a wide spectrum of cancers etoposide, doxorubicin, daunorubicin, and mitoxantrone [5, 7]. Both Topo I and II have an overlapping functions in DNA metabolism and essential in the normal progression of the cell cycle, so targeting both enzymes simultaneously lead to synergistic anticancer effects [5, 7] [62]. So, the dual activity of CPT as an efficient inhibitor of Topo I, II, in addition to its antifungal is one of the most intriguing biological criteria since the chemotherapy cause suppression to the immune system, permitting to the opportunistic microbial flora to be pathogenic. In cancer patients, invasive fungal disease remains an important complication causing considerable mortality and morbidity. The activity of CPT against *Rhizopus* sp is a very promising criterion, since *Rhizopus* is one of the causes of Mucormycosis, an emerging invasive fungal infection in immunocompromised patients [63]. So, this assumption was authenticated from the common properties of tumor and fungal cells such as replication rate, modalities of spreading within the host, rapid development of drug-resistance, and tendency to be more aggressive during disease progression [64].

The effect of extracted *A. terreus* CPT on the wound healing activity of the UO-31 was assessed, after 24 and 48 h, comparing to untreated cells. The wound healing of the monolayer cells of UO-31 was reduced by about 60%, comparing to control. The remarkable wound healing suppression ensures the interference of CPT with the cell regeneration, cell divisions, and matrix formation of the tumor cells UO-31. So, the strong antiproliferative activity of *A. terreus* CPT could be due to inhibition of Topo I and II, in addition to prevent the cellular matrix formation, and motility. The wound healing assay is a standard in vitro approach for checking the collective cell migration in two dimensions [48]. The cell migration is usually involved in several pathological disorders such as tumor invasion, angiogenesis, and metastasis [48, 65]. The apoptotic process of UO-31 in response to CPT of *A. terreus* was assessed by Annexin V- PI assay. A significant shift of the normal cells to apoptotic phase in response to *A. terreus* CPT, compared to control cells (untreated cells). Upon addition of *A. terreus* CPT, the total apoptosis of UO-31 cells was increased by 16 folds, compared to the untreated cells. The growth of UO-31 cells was maximally arrested at S-phase, compared to the control “untreated” cells. Similar results for CPT on apoptosis and cell cycle [66]

The productivity of CPT by *A. terreus* was maximized by Plackett–Burman nutritional optimization design, the actual yield of CPT was increased into 255.5 μg/L, at run #10, compared to the control cultures. Similar results for maximizing the CPT yield by *A. terreus*, *A. flavus*, and *P. chrysogenum* by Plackett–Burman Design bioprocessing were reported [21–25, 33]. So, the yield of *A. terreus* CPT was increased by ~ 2.5 folds, compared to the control cultures. The biosynthetic stability of *A. terreus* for CPT production with the subculturing and storage was assessed. The yield of CPT by the 1st culture of *A. terreus* was reduced by ~ 2.2 folds by the 5th generation, also, the CPT productivity by *A. terreus* was reduced by ~ 50%, by the 3rd month. So, attenuation of the CPT productivity by fungi with the subculturing and storage is the challenge that halts the further ongoing industrial uses of fungi to be CPT producing platform [22, 23, 29, 55]. Several hypotheses unravel the fungal-plant interactions, revealing the dependence of the biosynthetic machinery of CPT by the endophytic fungus on the some chemical signals from the plant or from their indigenous microbiome [22, 23, 25, 29, 55, 67, 68]. The biosynthetic potency of CPT by the 5th culture of *A. terreus* was not only restored, but also over-increased above the 1st culture of *A. terreus* by ~ 1.3 folds, in response to surface sterilized leaves of *C. roseus.* So, with the addition of leaves parts of *C. roseus*, the biosynthetic machinery of *A. terreus* CPT was reinstated, suggesting the releases of indigenous microbiome of plant tissues, microbiome cross-communication, and intimate growth with *A. terreus* triggering their CPT biosynthesis machinery [22, 23, 25, 29, 55, 67, 68].

In conclusion, *A. terreus,* an endophyte of *C. roseus,* was the potent CPT producers, the CPT had a strong activity toward the non CPT-producing fungal isolates, while, the CPT producing fungi gave an obvious resistance to CPT toxicity. The purified *A. terreus* CPT had a potential antiproliferative activity, inhibition of Topo I and II, preventing the wound healing, and induce the cellular apoptosis. The biosynthetic potency of *A. terreus* CPT was attenuated with the subculturing and storage, however, this biosynthetic machinery was completely restored upon addition of surface sterilized leaves of *C. roseus,* confirming the releases of specific signals from plant tissues or from their entire microbiome triggering the expression of biosynthetic machinery of *A. terreus* CPT. Further studies are ongoing to explore the molecular biosynthetic machinery of CPT by *A. terreus* with differential transcriptomics and proteomic approaches, to sustain their biosynthetic stability, to be a novel industrial platform for CPT production.

### Supplementary Information

**Additional file 1: Table S1.** Screening for the fungal endophytes from *Catharanthus roseus*.

## Data Availability

All datasets generated for this study are included in the article/additional file.
