# Supplementary material for: Camptothecin bioprocessing from Aspergillus terreus, an endophyte of Catharanthus roseus: antiproliferative activity, topoisomerase inhibition and cell cycle analysis
Source: Microb Cell Fact. 2024 Jan 5;23:15. doi: 10.1186/s12934-023-02270-4 (PMC10768243; doi:10.1186/s12934-023-02270-4)
Supplement: Supplementary file 1 — Additional file 1: Table S1. Screening for the fungal endophytes from Catharanthus roseus. [file 12934_2023_2270_MOESM1_ESM.docx]

**Table S1: Screening for the fungal endophytes from *Catharanthus roseus***

| Isolate  No. | Part | Fungi | Putative CPT yield on TLC (μg/l) | TLC  Visual | HPLC yield of CPT |
| --- | --- | --- | --- | --- | --- |
| 1 | Leaf | *Aspergillus awamori* | 0 | - | - |
| 2 | Twig | *Aspergillus niger* | 0 | - | - |
| 3 | Leaf | *Aspergillus fumigates* | 0 | - | - |
| 4 | Twig | *Pestalotia* sp | 67.64182 | ++ | 61.41125 |
| 5 | Twig | *Aspergillus flavus* | 0 | - | - |
| 6 | Flower | *Aspergillus restrictus* | 0 | - | - |
| 7 | Twig | *Penicillium polonicum* | 0 | - | - |
| 8 | Twig | *Aspergillus nidulans* | 0 | - | - |
| 9 | Twig | *Aspergillus flavipes* | 0 | - | - |
| 10 | Flower | *Aspergillus flavus* | 0 | - | - |
| 11 | Leaf | *Aspergillus tamari* | 0 | - | - |
| 12 | Leaf | *Cladosporium sp* | 0 | - | - |
| 13 | Flower | *Rhizopus* sp | 0 | - | - |
| 14 | Flower | *Aspergillus terreus* | 99.13108 | ++++ | 90 |
| 15 | Flower | *Aspergillus sydowi* | 0 | - | - |
| 16 | Flower | *Aspergillus glaucus* | 0 | - | - |
| 17 | Twig | *Alternaria* sp | 90.5402 | +++ | 82.20044 |
| 18 | Twig | *Chaetomium* sp | 0 | - | - |
| 19 | Twig | *Trichoderma spp.* | 38.56596 | + | 35.0136 |
| 20 | Flower | *Aspergillus tamarii* | 0 | - | - |
| 21 | Flower | *Aspergillus versicolor* | 27.32585 | + | 24.80-883 |
| 22 | Flower | *Penicillium* sp | 25.53174 | + | 23.17998 |
| 23 | Leaf | *Aspergillus* sp | 0 | - | - |
| 24 | Twig | *Alternaria brassicicola* | 94.564574 | +++ | 87.6699 |
| 25 | Twig | *Penicillium citrinum* | 0 | - | - |
